# Protective effect of uric acid against 6-OHDA-induced injury in SH-SY5Y cells

**DOI:** 10.1186/1750-1326-7-S1-S12

**Published:** 2012-02-07

**Authors:** Tingting Huang, Li Gong, Weifeng Luo, Lifang Hu, Chunfeng Liu

**Affiliations:** 1 The Department of Neurology, Second Affiliated Hospital of Soochow University, Suzhou,China; 2 The Institute of Neuroscience of Soochow University, Suzhou, China

## Objective

To investigate the effect of uric acid (UA) on 6-hydroxydopamine (6-OHDA) -induced injury in SH-SY5Y cells.

## Methods

The cell viability was measured by the MTT reduction assay. The cell apoptosis was assessed by Hoechst 33342 staining with fluorescence microscopy. The phophorylation of Akt and GSK-3β(ser9) was determined by Western blot analysis.

## Results

Treatment with 6-OHDA at 50µM for 12 h significantly decreased the viability of SH-SY5Y cells. Pretreatment with UA (200-400µM, 0.5h) prior to 6-OHDA treatment markedly increased the cell viability of SH-SY5Y cells, as compared to that of 6-OHDA-treated group. The beneficial effects of UA against 6-OHDA-induced apoptosis were also confirmed by Hoechst 33342 staining assay. Moreover, 6-OHDA decreased the Akt activity and increased the GSK-3β activity, which could be blocked by UA (200-400µM) pretreatment.

## Conclusions

These data suggest that 6-OHDA-induced cell injury was attenuated by UA. The underlying mechanisms may involve the up-regulation of Akt and the reduction of GSK-3β activity.

